# The potential of using data planning and analysis strategies to transform primary healthcare

**DOI:** 10.11606/s1518-8787.2023057supl3ed

**Published:** 2024-04-01

**Authors:** Alexandre D. P Chiavegato, Fernando P Cupertino de Barros

**Affiliations:** I Universidade de São Paulo Faculdade de Saúde Pública Departamento de Epidemiologia São Paulo SP Brasil Universidade de São Paulo. Faculdade de Saúde Pública. Departamento de Epidemiologia. São Paulo, SP, Brasil.; II Universidade Federal de Goiás Faculdade de Medicina Goiânia GO Brasil Universidade Federal de Goiás. Faculdade de Medicina. Goiânia, GO, Brasil.; III Conselho Nacional de Secretários de Saúde Brasília DF Brasil Conselho Nacional de Secretários de Saúde. Brasília, DF, Brasil

The fragmentation of healthcare is a reality for most countries. Over the last few decades, health systems have presented a natural trend towards decentralization, bringing important benefits by approximating public administration to local realities. However, this trend brought barriers for coordinating joint activities involving different levels of vertical and horizontal management.

Despite recent serious crises of infectious diseases, epidemiological research in Brazil show the increasing prevalence of chronic diseases, both in terms of their prevalence in the population and their impact on healthcare expenses. In this scenario, integrating medium and long-term healthcare, especially among older adult patients, will be an increasing challenge to ensure the sustainability of a health system such as the Brazilian one, which aims to be universal and comprehensive.

This supplement points to the possibility that the current decentralization trend can be accompanied by efficient planning of the health system for a more coordinated and equitable management of local resources. This planning aims to assist the development of skills and competencies of public administrators and healthcare professionals to organize and monitor health processes based on local needs, providing important contributions to work processes of health teams.

The first article of this supplement^1^) presents the challenges for implementing the PlanificaSUS, which was created by the Proadi-SUS project “*A Organização da Atenção Ambulatorial Especializada em Rede com a Atenção Primária à Saúde*,” implemented by the Hospital Israelita Albert Einstein, based on an innovative model, proposed and widely tested by the Brazilian National Council of Health Secretaries (CONASS – *Conselho Nacional de Secretários de Saúde*) over the last decade. The authors found that, despite advances, such as in the case of better articulation of networks and lines of care, important barriers persist, such as those related to the difficulty of regional planning articulated with specialized care.

Other studies in this supplement include: the analysis of changes in maternal and child healthcare practices in outpatient clinics that adopted PlanificaSUS^2^; the identification of previous knowledge in the mental health of higher education professionals^3^; the analysis of the implementation of a digital tool in primary healthcare^4^; among others.

The articles point to the potential of strategies for monitoring and organizing primary healthcare by analysis and data collection in various establishments. As the speed of the digital transformation of Brazilian health advances, there is great potential to improve the efficiency and equity of health management by planning and data analysis, allowing the correction of courses and the improvement of strategies.
