# Supplementary material for: The potential of using data planning and analysis strategies to transform primary healthcare
Source: Rev Saude Publica. 2024 Apr 1;57(Suppl 3):1s. doi: 10.11606/s1518-8787.2023057supl3ed (PMC11037910; doi:10.11606/s1518-8787.2023057supl3ed)
Supplement: Translation

# Translation: O potencial do uso de estratégias de planificação e análise de dados para transformar a atenção primária à saúde

**Authors:** Alexandre D. P Chiavegato, Fernando P Cupertino de Barros

**Affiliations:** I Brasil Universidade de São Paulo. Faculdade de Saúde Pública. Departamento de Epidemiologia. São Paulo, SP, Brasil.; II Brasil Universidade Federal de Goiás. Faculdade de Medicina. Goiânia, GO, Brasil.; III Brasil Conselho Nacional de Secretários de Saúde. Brasília, DF, Brasil

A fragmentação da atenção à saúde é uma realidade para a maioria dos países. Ao longo das últimas décadas, percebe-se um movimento natural em direção à descentralização dos sistemas de saúde, o que tem trazido benefícios importantes em relação à proximidade da gestão com as realidades locais. Por outro lado, esse movimento tem gerado novos desafios em relação à coordenação de atividades conjuntas entre os diferentes níveis de gestão vertical e horizontal.

Mesmo com as graves crises recentes de doenças infecciosas, o caminho epidemiológico do Brasil mostra o predomínio cada vez maior das doenças crônicas, tanto em termos da sua prevalência na população, como em relação à sua importância para o custo total da atenção à saúde. Nesse cenário, a integração dos cuidados de saúde de médio e longo prazo, principalmente entre pacientes idosos, será um desafio cada vez maior para garantir a sustentabilidade de um sistema de saúde como o brasileiro, que se propõe a ser universal e integral.

Este suplemento aponta para a possibilidade de o atual movimento de descentralização ser acompanhado por uma planificação eficiente do sistema de saúde, para, com isso, garantir uma gestão mais coordenada e equitativa dos recursos locais. A planificação tem como objetivo auxiliar no desenvolvimento de habilidades e competências por parte gestores e profissionais de saúde, para organizar e monitorar os processos da atenção à saúde com foco nas necessidades locais, aportando contribuições importantes na melhoria do processo de trabalho das equipes de saúde.

O primeiro artigo deste suplemento^1^ apresenta os desafios da implementação do PlanificaSUS, que foi criado pelo projeto Proadi-SUS “A Organização da Atenção Ambulatorial Especializada em Rede com a Atenção Primária à Saúde”, executado pelo Hospital Israelita Albert Einstein, a partir de um modelo inovador, proposto e amplamente testado pelo Conselho Nacional de Secretários de Saúde (Conass) ao longo da última década. Os autores descobriram que, apesar de avanços, como no caso da melhor articulação de redes e linhas de cuidado, ainda restam importantes barreiras, como aquelas que se referem à dificuldade do planejamento regional articulado com a atenção especializada.

Entre os outros estudos deste suplemento, está incluída a análise das mudanças de práticas do cuidado à saúde materno-infantil em ambulatórios que adotaram o PlanificaSUS^2^; a identificação do conhecimento prévio em saúde mental de profissionais de nível superior^3^; a análise da implementação de uma ferramenta digital na atenção primária à saúde^4^, entre outros.

Os artigos apontam para o potencial de estratégias de monitoramento e organização da atenção primária à saúde, por meio da análise e coleta de dados nos diferentes estabelecimentos. À medida em que a velocidade da transformação digital da saúde brasileira avança, existe o grande potencial de se melhorar a eficiência e equidade da gestão em saúde, por meio da planificação e análise de dados, permitindo a correção de rumos e o aperfeiçoamento de estratégias.
